# Stakeholders’ recommendations for revising Good Clinical Practice

**DOI:** 10.1016/j.conctc.2021.100776

**Published:** 2021-04-16

**Authors:** Amy Corneli, Annemarie Forrest, Teresa Swezey, Li Lin, Pamela Tenaerts

**Affiliations:** a Clinical Trials Transformation Initiative, Duke University, Durham, NC, USA; b Department of Population Health Sciences, Duke University School of Medicine, Durham, NC, USA; c Duke Clinical Research Institute, Duke University School of Medicine, Durham, NC, USA

**Keywords:** ICH E6 GCP, Clinical trials, Stakeholders, Good clinical practice, Ethics, Regulatory

## Abstract

The International Council for Harmonisation of Technical Requirements for Pharmaceuticals for Human Use (ICH) is revising ICH E6 Good Clinical Practice (GCP). The Clinical Trials Transformation Initiative (CTTI) initiated a project to identify and provide ICH with stakeholders’ priority areas and suggestions for revising ICH E6 GCP. We conducted a global online survey to identify areas of ICH E6 GCP that are and are not in need of revision. A total of 327 stakeholders completed the survey. Stakeholders represent many research roles and types of organizations, are employed in 39 countries, and conduct research in 153 countries. The ICH E6 GCP principles mentioned most often (range, 25%–29%) in need of revision were implementing systems that assure quality, providing medical care by qualified physicians/dentists, protecting confidentiality and privacy, obtaining informed consent, and documenting and storing information. The Investigator section (n = 244, 75%) and Sponsor section (n = 242, 74%) of ICH E6 GCP were identified as needing the most revision and the Investigator Brochure section (n = 166, 51%) as needing the least revision. The topic most frequently mentioned as needing revision is Monitoring (n = 146; 45%) in the Sponsor section. Although none of the principles or topics in ICH E6 GCP were identified as needing revision by the majority of stakeholders, a meaningful percentage of stakeholders identified areas that they believe need revision. These findings, which represent the views of a wide variety of stakeholders, may be useful to ICH for identifying where specifically to focus their revision efforts. CTTI provided the final report to ICH with the project findings for their consideration.

## 1 Introduction

The International Council for Harmonisation of Technical Requirements for Pharmaceuticals for Human Use (ICH) is currently revising ICH E6 Good Clinical Practice (GCP) [1]. The mission of ICH is to achieve worldwide harmonisation for developing safe, effective, and high quality medicines [2], and they have published numerous clinical guidelines to facilitate this mission, such as the ICH E6 GCP Guideline [1]. The ICH E6 GCP guideline serves as an “international ethical and scientific quality standard for designing, conducting, recording and reporting trials that involve the participation of human subjects” (ICH, 2016, page 1). Guideline compliance provides assurance to the public, according to ICH, that trial participants’ rights, safety and well-being are protected, and data collected are credible. ICH emphasizes that the E6 GCP guideline is intended for clinical trials that are conducted for regulatory submission [1]. ICH is revising their guidelines so that E6 GCP addresses diverse trial types and data sources and facilitates the use of technologies in trials [3,4].

The Clinical Trials Transformation Initiative (CTTI) independently initiated a multi-method project to identify areas in ICH E6 GCP that are of greatest need for revision and to describe stakeholder experiences with implementing ICH E6 GCP, including suggested ways to revise the guidance. CTTI is a public-private partnership cofounded by Duke University and the US Food and Drug Administration that seeks to develop and drive adoption of practices that will increase the quality and efficiency of clinical trials. We aimed to provide an opportunity for a diverse group of individuals who use ICH E6 GCP worldwide to share their views on how the guideline should be revised, addressing a criticism of the ICH guideline development process: lack of broad stakeholder engagement [5]. Our project consisted of 3 phases: (1) a global online survey, (2) qualitative, in-depth telephone interviews, and (3) an open comment platform [6].

The primary purpose of the survey was to identify areas of ICH E6 GCP that stakeholders believe are and are not in need of revision, thus highlighting the areas where revisions are needed the most. The follow-up qualitative interviews focused on gathering stakeholders’ experiences in implementing ICH E6 GCP as well as their suggestions for how the guideline should be revised. The open comment platform provided stakeholders an opportunity to provide line-by-line edits to the guideline. The full reports of the qualitative interviews and open comments can be found on the CTTI webpage [6]. A summarized version of the qualitative interviews will be published elsewhere. Here we describe the survey findings.

## 2 Methods

### 2.1 Study design

We conducted an on-line descriptive survey with stakeholders of ICH E6 GCP.

### 2.2 Recruitment

Organizations that have robust global professional research networks forwarded the survey invitation to their network members so we could reach a wide variety of stakeholders worldwide. These organizations were identified by CTTI leadership, internet searches, and the CTTI advisory group for this project, which included representatives from regulatory agencies, pharmaceutical companies, ethics review boards, contract research organizations, patient groups, clinical trials professional societies, academic institutions, and community-based health care organizations. Participating organizations forwarded a recruitment email to their members that included a link to the online survey and a request that recipients forward the recruitment email to others who might be interested in completing the survey. CTTI also posted the survey link via Twitter and LinkedIn. The initial response to the survey was limited in areas outside of North America and Europe; we therefore conducted a second wave of recruitment focusing on stakeholders who were part of research networks in ICH member countries, specifically Brazil, China, Republic of Korea, Japan, and Singapore, as well as research networks that conduct research in Africa. Data were collected from August 15 to September 20, 2019.

### 2.3 Eligibility

Stakeholders were eligible to complete the survey if they (1) self-reported that they are involved in research in a professional capacity, (2) have access to a computer and a reliable internet connection, and (3) read English; we offered the survey in English only, the official language of ICH.

### 2.4 Data collection

We purposefully created the survey to be short and targeted, keeping questions broad and focused on identifying priority areas for revision. We asked stakeholders to (1) review a list of the ICH E6 GCP principles and sections/topics and indicate whether they believe that the area is or is not in need of revision, or if they have no comments, and (2) answer demographic questions. Stakeholders reflected on the ICH E6 GCP R2 addendum [1].

### 2.5 Data analysis

We used descriptive statistics to summarize the data and describe the survey findings.

### 2.6 Ethics

The project was reviewed and determined exempt from research oversight by the Duke University Health System Institutional Review Board.

## 3 Results

### 3.1 Study population

Of the 737 stakeholders who initiated the survey, 327 responded to all questions and were included in the final sample. The most common type of research conducted by stakeholders (n=289; 88%) was phase I, II, or III clinical research on medicinal products (drugs, vaccines, and biologicals) (Table 1). Stakeholders were from 39 countries representing every region of the world, although most stakeholders’ places of employment were in Europe and Central Asia (n = 193; 60%), primarily European countries, and North America (n = 98; 31%) (eAppendix 1, Table 1). Stakeholders conducted research in 153 countries worldwide (eAppendix 1, Table 2), were affiliated with a wide range of organizational types (eAppendix 1, Table 3), and represented many research roles (eAppendix 1, Table 4); 79% (n = 259) had 10 or more years of experience in research (eAppendix 1, Table 5), nearly all (n = 304; 93%) received training on ICH E6 GCP, and most (n = 258; 79%) regularly relied on ICH E6 GCP in their research role (eAppendix 1, Table 6).

**Table 1:** Type of research stakeholders conduct (current and past).

Type of Research	No. (%)^a^
Phase I, II, or III clinical research on medicinal products (drugs, vaccines, and biologicals)	289 (88.4)
Observational clinical research	186 (56.9)
Phase IV: Post-marketing/post-approval clinical research on medicinal products	182 (55.7)
Epidemiological research	112 (34.3)
Other clinical research not on medicinal products	74 (22.6)
Diagnostic studies	73 (22.3)
Other clinical research on medicinal products	68 (20.8)
Social science and behavioral research	46 (14.1)

a Stakeholders selected all that applied.

### 3.2 Stakeholder recommendations for revising the principles of ICH E6 GCP

**Fig. 1:**
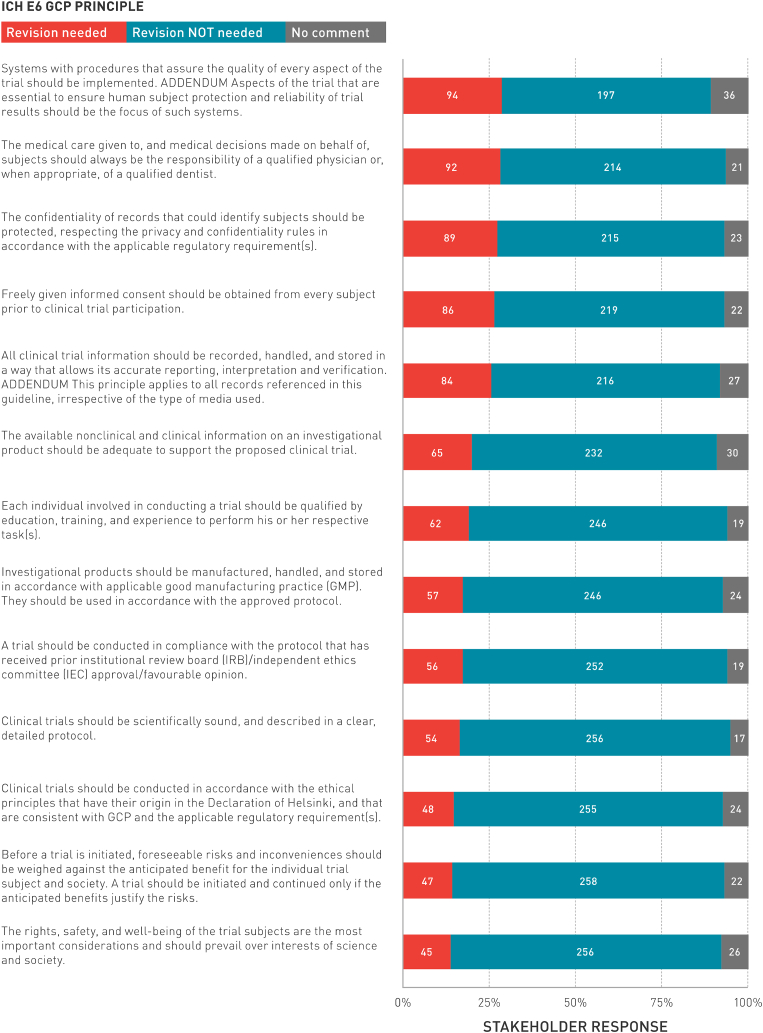
Stakeholder recommendations for revising the ICH E6 GCP principles.

Fig. 1 presents stakeholder's recommendations for revising the ICH E6 GCP principles, ranked in order of need. The 5 principles most commonly identified by stakeholders in need of revision are:

1. Implementing systems that assure quality (n = 94; 29%)
2. Providing medical care by a qualified physician or dentist (n = 92; 28%)
3. Protecting the confidentiality of participant records and privacy (n = 89; 27%)
4. Obtaining informed consent (n = 86; 26%)
5. Documenting and storing clinical trial information to ensure accurate reporting, interpretation and verification (n = 84; 25%)

The 5 most common principles stakeholders believed did not need revision are:

1. Weighing risks and benefits (n = 258; 79%)
2. Considering the rights, safety, and well-being of the trial subjects over interests of science and society (n = 256; 78%)
3. Implementing scientifically sound clinical trials with a clear, detailed protocol (n = 256; 78%)
4. Conducting clinical trials in accordance with the ethical principles and GCP (n = 255; 78%)
5. Obtaining approval from an independent ethics committee approval (n = 252; 77%)

### 3.3 Stakeholder recommendations for revising the content of ICH E6 GCP

**Fig. 2:**
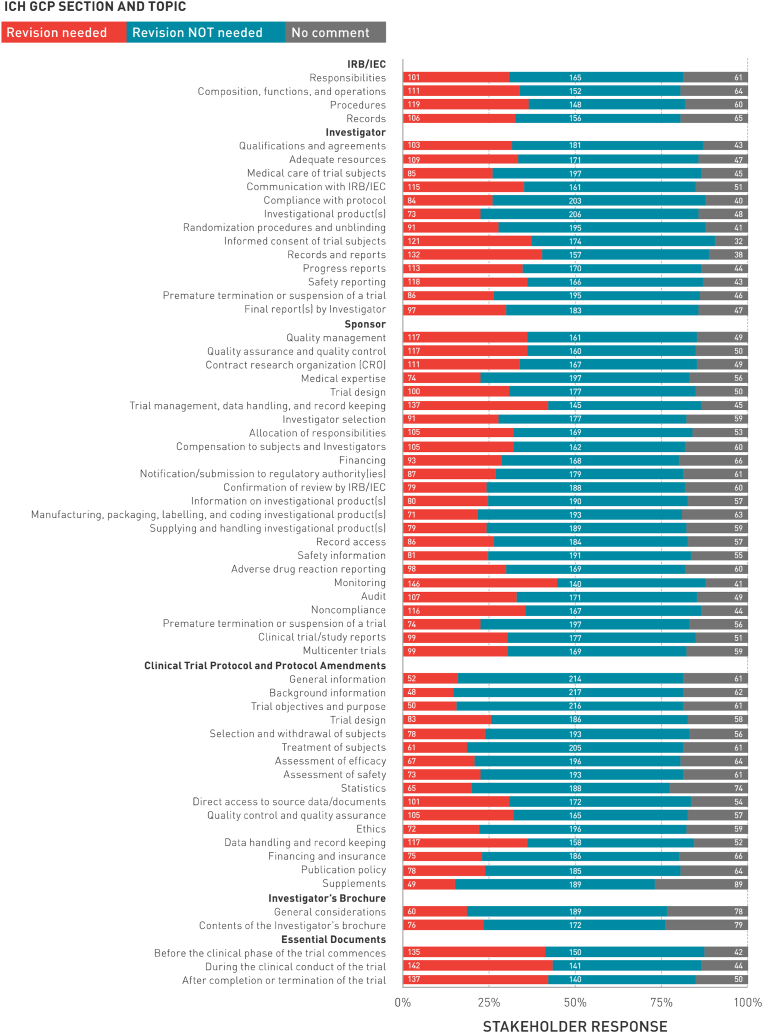
Stakeholder recommendations for revising the ICH E6 GCP topics by section.

The sections in most need of revision, based on the number of stakeholders indicating that at least one topic in that section should be revised, are the Investigator (n = 244, 75%) and Sponsor (n = 242, 74%) sections. Fig. 2 presents stakeholder's recommendations for revising the ICH E6 GCP topics, by section. The top 5 topics reported in need of revision are:

1. Sponsor: Monitoring (n = 146; 45%)
2. Essential Documents: During the Clinical Conduct of the Trial (n = 142; 43%)
3. Essential Documents: After Completion or Termination of the Trial (n = 137; 42%)
4. Sponsor: Trial Management, Data Handling, and Record Keeping (n = 137; 42%)
5. Essential Documents: Before the Clinical Phase of the Trial Commences (n = 135; 41%)

The section in least need of revision, based on the number of stakeholders indicating that no topic in that section should be revised, is the section on the Investigator Brochure (n = 166, 51%). The top 5 topics that stakeholders reported not needing revision are:

1. Clinical Trial Protocol and Protocol Amendments: Background Information (n = 217; 66%)
2. Clinical Trial Protocol and Protocol Amendments: Trial Objectives and Purpose (n = 216; 66%)
3. Clinical Trial Protocol and Protocol Amendments: General Information (n = 214; 65%)
4. Investigator: Investigational Product(s) (n = 206; 63%)
5. Clinical Trial Protocol and Protocol Amendments: Treatment of Subjects (n = 205; 63%)

## 4 Discussion

We aimed to identify aspects of ICH E6 GCP that stakeholders believed needed to be revised—and not revised—based on their experiences in implementing the guideline. Overall, none of the principles or topics in ICH E6 GCP were identified as needing revision by the majority of stakeholders. However, a meaningful percentage of stakeholders identified areas that they believe need revision. Of the 6 sections of ICH E6 GCP, the Sponsor and Investigator sections were identified as needing the most revision, although topics from other sections were also identified as needing revision. Of less need for revision was the Investigator Brochure section, although stakeholders identified topics from other sections that were also in less need of revision.

A strength of the research is that we describe the views of a wide variety of stakeholders who represent multiple countries, a diversity of research roles, and numerous types of organizations. However, even with significant effort, we had limited stakeholder involvement in areas outside of North America and Europe. Offering the survey in English only, the official language of ICH, may have been a barrier. In addition, as with all descriptive research, the findings presented here represent the views of the individuals who participated; a different group of individuals could have yielded different findings. Lastly, to facilitate ease of completion, we solicited stakeholders’ feedback within the current familiar organizational structure of ICH E6 GCP; a different format may have yielded different findings.

CTTI provided the final report [6] to ICH for their consideration as they were revising ICH E6 GCP, although CTTI has no agreement with ICH that they will use the findings in their planned revision. We hope these data will be useful to ICH to determine where specifically to focus their revision efforts.

## Funding

Funding for this work was made possible, in part, by the 10.13039/100000038US Food and Drug Administration through cooperative agreement U18FD005292 and grant R18FD005292. Views expressed in written materials or publications and by speakers and moderators do not necessarily reflect the official policies of the Department of Health and Human Services, nor does any mention of trade names, commercial practices, or organization imply endorsement by the United States Government. Partial funding was also provided by pooled membership fees from the Clinical Trials Transformation Initiative's (CTTI) member organizations.

## Declaration of competing interest

We wish to confirm that there are no known conflicts of interest associated with this publication and there has been no significant financial support for this work that could have influenced its outcome.

## Figures and Tables

**Table 1:** Geographic Location of Stakeholders' Employment (n = 321*)

Region/Country	No. (%)
**East Asia and Pacific**	**21 (6.5)**
Australia	16 (5.0)
China	1 (0.3)
Japan	4 (1.2)
**Europe and Central Asia**	**193 (60.1)**
Austria	3 (0.9)
Belgium	6 (1.9)
Bulgaria	1 (0.3)
Croatia	1 (0.3)
Czechia (Czech Republic)	4 (1.2)
Denmark	5 (1.6)
Estonia	2 (0.6)
Finland	4 (1.2)
France	7 (2.2)
Germany	56 (17.4)
Greece	3 (0.9)
Hungary	2 (0.6)
Ireland	10 (3.1)
Italy	9 (2.8)
Netherlands	7 (2.2)
Norway	4 (1.2)
Poland	1 (0.3)
Portugal	5 (1.6)
Romania	3 (0.9)
Serbia	2 (0.6)
Slovakia	3 (0.9)
Slovenia	3 (0.9)
Spain	9 (2.8)
Sweden	4 (1.2)
Switzerland	11 (3.4)
Turkey	1 (0.3)
United Kingdom	27 (8.4)
**Latin America and Caribbean**	**3 (0.9)**
Argentina	1 (0.3)
Colombia	2 (0.6)
**Middle East and North Africa**	**4 (1.2)**
Iraq	1 (0.3)
Israel	2 (0.6)
Lebanon	1 (0.3)
**North America**	**98 (30.5)**
Canada	48 (15.0)
United States of America	50 (15.6)
**South Asia**	**1 (0.3)**
Sri Lanka	1 (0.3)
**Sub-Saharan Africa**	**1 (0.3)**
Tanzania	1 (0.3)

*Data are missing from 6 stakeholders.

**Table 2:** Geographic Location of Stakeholders' Research^a^

Country	No. (%)
**East Asia and Pacific**	**97 (29.7)**
Australia	72 (22.0)
Cambodia	3 (0.9)
China	40 (12.2)
Indonesia	14 (4.3)
Japan	41 (12.5)
Laos	1 (0.3)
Malaysia	18 (5.5)
Maldives	1 (0.3)
Marshall Islands	1 (0.3)
Mongolia	1 (0.3)
Myanmar	3 (0.9)
New Zealand	47 (14.4)
Papua New Guinea	1 (0.3)
Philippines	16 (4.9)
Singapore	27 (8.3)
South Korea	35 (10.7)
Taiwan	30 (9.2)
Thailand	27 (8.3)
Vietnam	12 (3.7)
**Europe and Central Asia**	**230 (70.3)**
Albania	4 (1.2)
Andorra	3 (0.9)
Armenia	2 (0.6)
Austria	69 (21.1)
Azerbaijan	3 (0.9)
Belarus	12 (3.7)
Belgium	78 (23.9)
Bosnia and Herzegovina	6 (1.8)
Bulgaria	30 (9.2)
Croatia	26 (8.0)
Cyprus	8 (2.4)
Czechia (Czech Republic)	55 (16.8)
Denmark	71 (21.7)
Estonia	27 (8.3)
Finland	50 (15.3)
France	89 (27.2)
Georgia	8 (2.4)
Germany	134 (41.0)
Greece	39 (11.9)
Greenland	2 (0.6)
Hungary	51 (15.6)
Iceland	14 (4.3)
Ireland	73 (22.3)
Italy	93 (28.4)
Kazakhstan	3 (0.9)
Kosovo	2 (0.6)
Kyrgyzstan	3 (0.9)
Latvia	21 (6.4)
Liechtenstein	3 (0.9)
Lithuania	25 (7.6)
Luxembourg	11 (3.4)
Malta	4 (1.2)
Moldova	9 (2.8)
Monaco	3 (0.9)
Montenegro	1 (0.3)
Netherlands	80 (24.5)
North Macedonia (Formerly Macedonia)	3 (0.9)
Norway	53 (16.2)
Poland	69 (21.1)
Portugal	45 (13.8)
Romania	40 (12.2)
Russia	48 (14.7)
Serbia	18 (5.5)
Slovakia	31 (9.5)
Slovenia	23 (7.0)
Spain	80 (24.5)
Sweden	73 (22.3)
Switzerland	69 (21.1)
Tajikistan	1 (0.3)
Turkey	29 (8.9)
Turkmenistan	1 (0.3)
Ukraine	32 (9.8)
United Kingdom	112 (34.3)
Uzbekistan	1 (0.3)
Vatican City	1 (0.3)
**Latin America and Caribbean**	**59 (18.0)**
Argentina	39 (11.9)
Bahamas	1 (0.3)
Barbados	1 (0.3)
Belize	1 (0.3)
Bolivia	2 (0.6)
Brazil	38 (11.6)
Chile	27 (8.3)
Colombia	21 (6.4)
Costa Rica	4 (1.2)
Cuba	3 (0.9)
Dominica	1 (0.3)
Dominican Republic	3 (0.9)
Ecuador	7 (2.1)
El Salvador	2 (0.6)
Guatemala	5 (1.5)
Guyana	1 (0.3)
Honduras	1 (0.3)
Jamaica	2 (0.6)
Mexico	38 (11.6)
Nicaragua	1 (0.3)
Panama	5 (1.5)
Paraguay	4 (1.2)
Peru	11 (3.4)
Uruguay	5 (1.5)
Venezuela	10 (3.1)
**Middle East and North Africa**	**42 (12.8)**
Algeria	2 (0.6)
Bahrain	2 (0.6)
Egypt	10 (3.1)
Iran	1 (0.3)
Iraq	1 (0.3)
Israel	30 (9.2)
Jordan	4 (1.2)
Kuwait	2 (0.6)
Lebanon	6 (1.8)
Morocco	2 (0.6)
Oman	1 (0.3)
Qatar	5 (1.5)
Saudi Arabia	8 (2.4)
Syria	1 (0.3)
Tunisia	4 (1.2)
United Arab Emirates	6 (1.8)
Yemen	3 (0.9)
**North America**	**170 (52.0)**
Canada	119 (36.4)
United States of America	122 (37.3)
**South Asia**	**44 (13.5)**
Afghanistan	1 (0.3)
Bangladesh	6 (1.8)
India	43 (13.1)
Nepal	2 (0.6)
Pakistan	6 (1.8)
Sri Lanka	5 (1.5)
**Sub-Saharan Africa**	**49 (15.0)**
Benin	2 (0.6)
Botswana	1 (0.3)
Burkina Faso	1 (0.3)
Cameroon	4 (1.2)
Central African Republic (CAR)	1 (0.3)
Congo, Democratic Republic of the	3 (0.9)
Cote d'Ivoire	2 (0.6)
Ethiopia	4 (1.2)
Gabon	1 (0.3)
Gambia	4 (1.2)
Ghana	8 (2.4)
Guinea	1 (0.3)
Kenya	13 (4.0)
Liberia	1 (0.3)
Malawi	4 (1.2)
Mali	1 (0.3)
Mozambique	3 (0.9)
Namibia	2 (0.6)
Nigeria	6 (1.8)
Rwanda	4 (1.2)
Senegal	2 (0.6)
Seychelles	1 (0.3)
Sierra Leone	3 (0.9)
South Africa	37 (11.3)
Sudan	3 (0.9)
Tanzania	9 (2.8)
Uganda	10 (3.1)
Zambia	5 (1.5)
Zimbabwe	5 (1.5)

^a^The regional headers represent the total number and percentage of stakeholders who conduct research in one of the countries in that region. The country sub-headers represent the total number and percentage of stakeholders who conduct research in that country.

**Table 3:** Stakeholders' Affiliations (n = 327)

Organization Type	No. (%)
University/academic research center affiliated with a hospital/medical center	132 (40.4)
Pharmaceutical company or biotechnology company	61 (18.7)
Contract research organization (commercial/for profit)	44 (13.5)
Hospital/medical center not affiliated with a university/academic research center	21 (6.4)
University/academic research center not affiliated hospital/medical center	20 (6.1)
Governmental organization that regulates medical products	14 (4.3)
Non-governmental organization or not-for-profit organization	12 (3.7)
Private research site	8 (2.4)
Governmental organization that does not regulate medical products	5 (1.5)
Patient advocacy group	2 (0.6)
Private foundation	2 (0.6)
Trade/professional organization	1 (0.3)
Not affiliated with a specific organization	1 (0.3)
Prefer not to respond	4 (1.2)

**Table 4:** Stakeholders' Main Role in Research (n = 327)

Research Role	No. (%)
Principal investigator, co-investigator, sub-investigator, site investigator	77 (23.5)
Quality assurance/quality control personnel	65 (19.9)
Clinical operations personnel	57 (17.4)
Clinical research associate/research coordinator/study nurse	45 (13.8)
Regulatory affairs personnel	21 (6.4)
Data analyst	12 (3.7)
Monitor	10 (3.1)
Inspector	9 (2.8)
Data manager	6 (1.8)
Government regulator	4 (1.2)
Laboratory personnel	4 (1.2)
Pharmacist	3 (0.9)
Ethics review/Institutional Review Board personnel	1 (0.3)
Medical provider (healthcare provider who delivers medical care to study participants)	1 (0.3)
Data collector	1 (0.3)
Patient advocate	1 (0.3)
Prefer not to respond	10 (3.1)

**Table 5:** Length of Time Stakeholders Have Been Involved in Research (n = 327)

Length of Time	No. (%)
Less than 1 year	2 (0.6)
1 year to less than 5 years	24 (7.3)
5 years to less than 10 years	38 (11.6)
10 years to less than 20 years	108 (33.0)
20 or more years	151 (46.2)
Prefer not to respond	4 (1.2)

**Table 6:** Stakeholders' Engagement with ICH GCP E6 (n = 327)

Type of Engagement	No. (%)
Received training on ICH GCP	304 (93.0)
How often rely on GCP to do research role	
Regularly	258 (78.9)
Occasionally	41 (12.5)
Rarely	15 (4.6)
Never	2 (0.6)
Prefer not to respond	11 (3.4)
